# VDJ-Insights: simplifying the annotation of genomic immunoglobulin and T cell receptor regions

**DOI:** 10.1093/bioinformatics/btag108

**Published:** 2026-03-09

**Authors:** Susan E Ott, Giang N Le, Sayed J Mohammadi, Jesse Mittertreiner, Erica M Pasini, Ronald E Bontrop, Natasja G de Groot, Jesse Bruijnesteijn

**Affiliations:** Biomedical Primate Research Centre, Rijswijk 2288 GJ, The Netherlands; Biomedical Primate Research Centre, Rijswijk 2288 GJ, The Netherlands; Biomedical Primate Research Centre, Rijswijk 2288 GJ, The Netherlands; Biomedical Primate Research Centre, Rijswijk 2288 GJ, The Netherlands; Biomedical Primate Research Centre, Rijswijk 2288 GJ, The Netherlands; Biomedical Primate Research Centre, Rijswijk 2288 GJ, The Netherlands; Biomedical Primate Research Centre, Rijswijk 2288 GJ, The Netherlands; Biomedical Primate Research Centre, Rijswijk 2288 GJ, The Netherlands

## Abstract

**Motivation:**

Accurate annotation of germline immunoglobulin (IG) and T cell receptor (TCR) loci is critical for understanding adaptive immunity.

**Results:**

VDJ-Insights provides a user-friendly software package for characterizing these complex immune regions. In addition, it assesses gene segment functionality, identifies recombination signal sequences, and annotates complementarity-determining regions 1 and 2. VDJ-Insights achieved over 99% concordance with curated annotations from multiple species, outperforming existing annotation tools. When applied to 95 haplotypes from the Human Pangenome Reference Consortium, VDJ-Insights identified 652 and 275 novel IG and TCR alleles, respectively, highlighting its scalability for large immunogenetic studies.

**Availability and implementation:**

Datasets and software package are available in the VDJ-insights repository, https://github.com/BPRC-Bioinfo and https://doi.org/10.5281/zenodo.17588835. Additional intermediate datasets used and analyzed during the current study are available from the corresponding authors upon reasonable request.

## 1 Introduction

B cells and T cells are essential components of the adaptive immune system, with their respective receptors, the immunoglobulin receptor (IG) and the T cell receptor (TCR), serving as direct or indirect mediators of pathogen recognition. Genetically, these receptors are constructed in a similar fashion, involving the somatic rearrangement of variable (V), diversity (D), and joining (J) gene segments during the development of B and T cells. In B cells, this rearrangement takes place within the heavy (IGH) and light (IGK and IGL) chain regions, while in T cells it occurs across the TRA, TRB, TRG, and TRD regions (Early *et al.* 1980, Davis and Bjorkman 1988). These genomic regions exhibit substantial diversity, characterized by large structural variations, including deletions, insertions, and duplications of functional segments, as well as by allelic polymorphism (Kidd *et al.* 2012, Rodriguez *et al.* 2020, Rodriguez *et al.* 2022, Gibson *et al.* 2023, Rodriguez *et al.* 2023, Engelbrecht *et al.* 2024). The overall diversity of human IG and TCR gene segments is well documented, with 1069 and 1565 human V, D, and J alleles catalogued in databases such as International IMmunoGeneTics Information System (IMGT) and VDJbase (both assessed on 30 April 2025), respectively (Giudicelli *et al.* 2006, Omer *et al.* 2020). However, the individual haplotypes of these loci remain largely unexplored due to their complex organization, which hampers assembly, phasing, and annotation using conventional high-throughput methods and thus prevents routine documentation (Watson *et al.* 2013, Cirelli *et al.* 2019).

Understanding and documenting the diversity in these highly complex immune regions is of importance to elucidate the impact of germline variation on antibody and T cell responses. For example, specific IG gene segments have been linked to differential responses to vaccines, including those for malaria and seasonal influenza (Avnir *et al.* 2014, 2016, Martin *et al.* 2023, McDaniel et al. 2025). Furthermore, polymorphisms in IGH genes affect the function of neutralizing antibodies in response to HIV and SARS-CoV-2 infection (West *et al.* 2012, Scharf *et al.* 2013, Pushparaj *et al.* 2023). In the context of disease and vaccine associations, the IG and TCR loci in biomedically important model species, such as mice and macaques, have also been characterized, though only to a limited extent (Brekke and Garrard 2004, Retter *et al.* 2007, Greenaway *et al.* 2009, Yu *et al.* 2016, Ramesh *et al.* 2017, Cirelli *et al.* 2019, Nguefack Ngoune *et al.* 2022). Despite studies across multiple species and the extensive cataloging of human germline alleles in various databases, new sequences continue to be discovered (Vazquez Bernat *et al.* 2021, Gibson *et al.* 2023, Rodriguez *et al.* 2023, Debbagh *et al.* 2024, Engelbrecht *et al.* 2024). This indicates that even greater diversity is likely to emerge as more individuals and species are examined for their IG and TCR loci. Moreover, while most research to date has focused on coding regions, the noncoding sequences, including the recombination signal sequences (RSS), remain largely unexplored. RSS are relatively conserved DNA motifs flanking gene segments and guide their rearrangement. Overall, these noncoding features may be essential for the rearrangement processes that ultimately shape IG and TCR repertoires (Hesse *et al.* 1989, Hirokawa *et al.* 2020, Hoolehan *et al.* 2022).

With the introduction of long-read sequencing technologies, such as Pacific Biosciences (PacBio) and Oxford Nanopore Technologies (ONT) platforms, the characterization of novel genomic IG and TCR regions has become more accessible (Gibson *et al.* 2023, Rodriguez *et al.* 2023, Engelbrecht *et al.* 2024). Yet, their assembly and annotation remain time-consuming and require expert curation. To address these challenges, several bioinformatic tools, including gAIRR Suite, IGDetective, and Digger, have been developed to analyze either individual V, D, and J gene segments or the complete genomic IG and TCR regions (Lin *et al.* 2022, Sirupurapu *et al*. 2022, Lees *et al.* 2024). Among these, gAIRR Suite is specifically designed for annotating short-read assemblies, whereas IGDetective identifies gene segments without relying on a predefined segment library (Lin *et al.* 2022, Sirupurapu *et al*. 2022). The most recently published tool, Digger, enables identification of both known and novel gene segments while also predicting their functionality based on IMGT definitions (Lees *et al.* 2024). Building on these advancements, we developed VDJ-Insights, a tool that integrates and enhances similar features by automating the identification and annotation of both coding segments and noncoding elements. Additionally, it dynamically integrates the latest IMGT database updates and utilizes IMGT standards to predict functionality of known and newly identified gene segments. VDJ-Insights also assesses the RSS and annotates complementarity-determining regions (CDR) 1 and 2. These latter regions represent hypervariable loop sequences that, together with CDR3, form the antigen-binding site of antibodies and TCRs (Giudicelli and Lefranc 2012, Wong *et al.* 2019). The tool supports multi-sample processing and provides a user-friendly web-based interface for data interpretation, visualization, and comparative analysis.

We validated VDJ-Insights by analyzing IMGT-curated human sequences, as well as curated sequences from other species such as gorillas, rhesus macaques, and mice (Guo *et al.* 2017, Tran et al. 2021, Nguefack Ngoune *et al.* 2022, Debbagh *et al.* 2024). Following successful validation, samples from the Human Pangenome Reference Consortium (HPRC) were analyzed (Liao *et al.* 2023). This analysis resulted in the identification of 652 novel IG and 275 novel TCR V, D, and J alleles, of which 205 IG and 137 TCR were confirmed in at least two individuals. Collectively, these findings highlight the strength of VDJ-Insights in processing large-scale datasets and advancing the identification of both known and novel IG and TCR segments.

## 2 Materials and methods

### 2.1 Overview of the VDJ-Insights workflow

**Figure 1:**
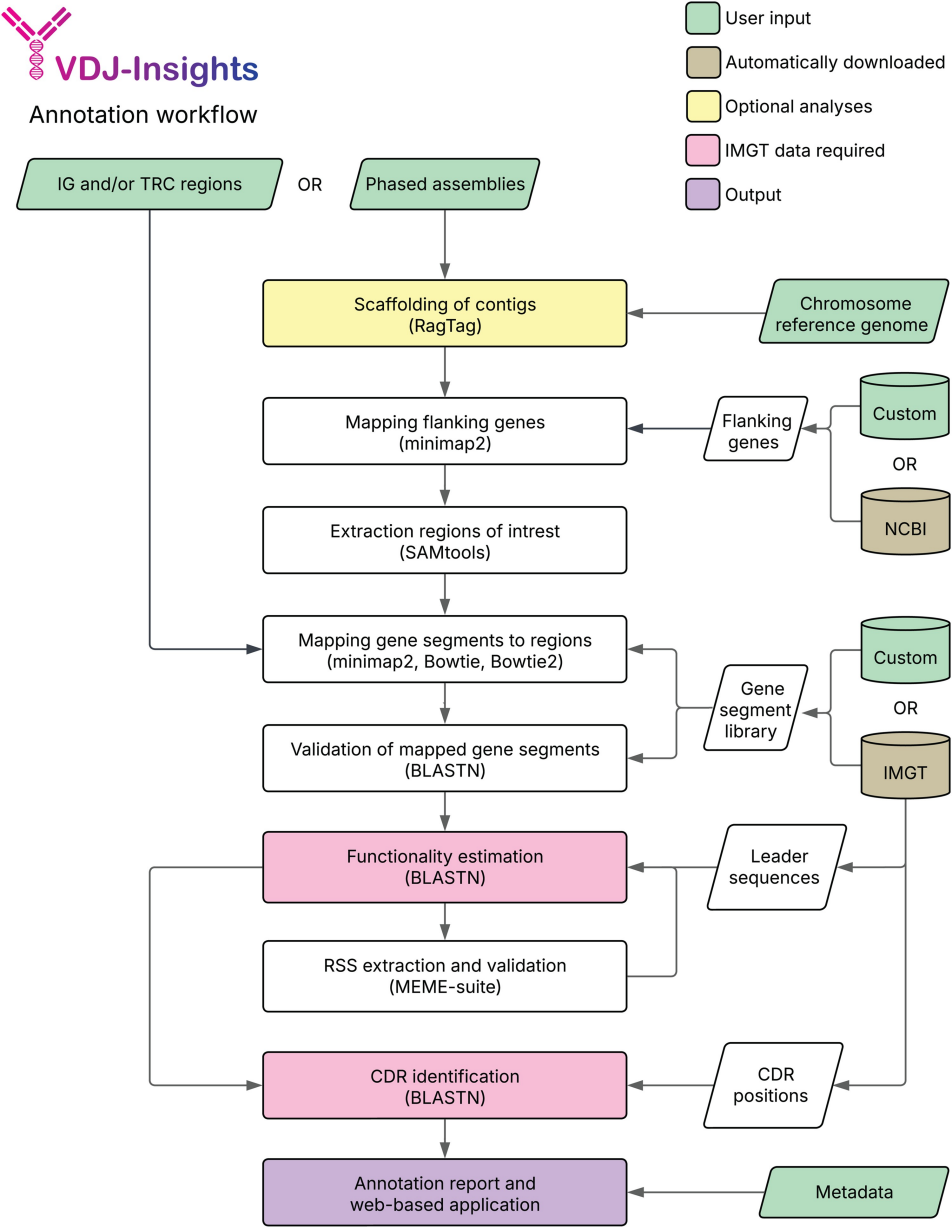
Workflow of the VDJ-Insights IG and TCR annotation. Different colors indicate user-provided input, input automatically retrieved by VDJ-Insights, optional analyses, and analyses requiring IMGT data. For each step, the primary tools used are listed in parentheses.

VDJ-Insights is developed to extract, annotate, and analyze the IG and TCR regions from genomic sequences (Fig. 1). First, VDJ-Insights extracts the IG or TCR regions using predefined flanking genes, which serve as markers for identifying the boundaries of the regions of interest. These flanking genes are relatively conserved among species. Our tool provides default flanking genes for humans and commonly used model species (e.g. rhesus macaque (*Macaca mulatta*) and house mouse (*Mus musculus*); Table 1, available as supplementary data at *Bioinformatics* online) but also allowing users to define custom flanking genes whenever needed. In cases where an immune region appears fragmented, thus with flanking genes located on distinct contigs, users can initiate an additional scaffolding step within the pipeline to potentially construct complete IG and/or TCR regions (Alonge *et al.* 2022). Next, a sequence library containing previously reported V, D, and J gene segments is mapped to the extracted regions, using Bowtie, Bowtie2, and minimap2 (Camacho *et al.* 2009, Langmead *et al.* 2009, Langmead and Salzberg 2012, Chen *et al.* 2015, Li 2021). When no library is provided by the user, VDJ-Insights automatically retrieves a gene segment library for the specified species from the IMGT database, if available (Giudicelli *et al.* 2006). Subsequently, mapped gene segments are filtered and evaluated using BLASTN and BTOP (Camacho *et al.* 2009, Chen *et al.* 2015). To predict gene functionality, VDJ-Insights examines multiple IMGT-based criteria, including the assessment of leader sequences, when available, the presence of start codons and the screening for in-frame stop codons (Table 1) (Lefranc 1996, https://www.imgt.org/IMGTScientificChart/SequenceDescription/IMGTfunctionality.html). Next, VDJ-Insights extracts the RSS flanking each segment. The RSS from segments preliminary classified as functional are built into motif models. Subsequently, FIMO is used to refine functionality predictions based on these RSS motifs. In addition to the RSS, VDJ-Insights identifies germline-encoded CDR1 and CDR2 by alignment to their IMGT reference sequences when available. As CDR3 is shaped through somatic recombination, it is currently not included in the VDJ-Insights analysis. The parameter settings are detailed in the extended methods (Methods, available as supplementary data at *Bioinformatics* online).

**Table 1:** Functionality criteria for V, D, and J gene segments.^a^

Gene segment	Functionality criteria	If criterion is not met
V	Leader sequences identified	Pseudogene
	Reading frame is a multiple of 3 base pairs	Pseudogene
	Start codon present	Pseudogene
	No in-frame stop codons	Pseudogene
	Stop codon at the end of the sequence	ORF
	Donor splice site (GT) present	ORF
	Acceptor splice site (AG) present	ORF
	Minimum two cysteines present	ORF
	Functional RSS motif present	Pseudogene
D	Functional RSS motifs present	Pseudogene
J	Motifs ([FW]G.G) present	ORF
	No premature stop codon present	Pseudogene
	Donor splice site (GT) present	Pseudogene
	Functional RSS motif present	Pseudogene

a Gene segment functionality is classified into three categories: functional, open-reading frame (ORF), and pseudogene. Gene segments designated as functional meet all criteria for expression and V(D)J rearrangement. Those classified as ORFs exhibit deviations in rearrangement or splicing signals but maintain an in-tact ORF. Pseudogenes are defined by the presence of frameshift mutations or premature stop codons that disrupt the ORF. These functionality classifications are predicted based on genomic features and are not supported by experimental data.

The output of VDJ-Insights includes a comprehensive overview of the identified known and novel gene segments, their predicted functionality, and an assessment of the RSS, CDR1, and CDR2 sequences, which are compiled into an interactive web-based annotation report. This report facilitates downstream analyses of the extracted IG and TCR regions, including the comparison of gene segments across multiple samples and the evaluation of newly identified sequences.

### 2.2 Validation and HPRC datasets

The annotation accuracy of VDJ-Insights was validated using expert-curated immunogenetic reference sequences from the IMGT database (Table 2, available as supplementary data at *Bioinformatics* online). Here, “accuracy” is defined as the proportion of annotations that match the corresponding curated IMGT annotations. This benchmarking set included five human IG and TR loci from the GRCh38 human reference genome (Guo *et al.* 2017), three IG regions from four Western lowland gorilla (*Gorilla gorilla*) genome assemblies (Debbagh *et al.* 2024), IG regions from the rhesus macaque reference genome (Nguefack Ngoune *et al.* 2022), and the IGH and IGL loci of four laboratory house mouse strains, including C57BL/6J, BALB/cJ, DBA/2J, and NOD/SCID (Table 2, available as supplementary data at *Bioinformatics* online) (Tran et al. 2021). Gene segment annotations and predicted functionality generated by VDJ-Insights were systematically compared to these IMGT-curated references using a custom validation script, which is publicly available via GitHub.

Following validation, the tool was applied to analyze a comprehensive dataset of 95 haploid genome assemblies to demonstrate its scalability. This included the initial batch of genomes released by the HPRC (release 1), many of which were generated using DNA derived from Epstein–Barr virus transformed lymphoblastoid cell lines (LCLs) (Liao *et al.* 2023). Because LCL-derived assemblies can contain somatic rearrangements in IG regions, annotations of these regions should be interpreted with caution (Rodriguez *et al*. 2021). To mitigate this, novel IG alleles identified in LCL samples were required to be present in at least four individuals. A similar cautious approach to annotate IG regions from LCL-derived short-read assemblies was used in a previous study (Khatri *et al.* 2021). Although that study included a larger sample size, which strengthens their confirmation of novel alleles, we consider the HPRC assemblies to be high quality, particularly given the use of long reads and a trio binning strategy (Liao *et al.* 2023). TCR regions are likely less affected by the LCL origin. In addition to the HPRC assemblies, we included the most recent telomere-to-telomere (T2T) assembly, which is not derived from a B cell line (Guo *et al.* 2017). We processed the complete set of assemblies in one batch for the IG regions and one batch for the TCR regions, leveraging the parallel computing capabilities of VDJ-Insights. Sample metadata corresponding to the HPRC assemblies were retrieved from the HPRC Data Explorer (https://data.humanpangenome.org/assemblies).

### 2.3 Annotating V, D, and J gene segments using Digger

To further benchmark VDJ-Insights, all IMGT-curated sequences from human, Western lowland gorilla, rhesus macaque, and house mouse were also analyzed using Digger (v0.7.5) (Lees *et al.* 2024). To support RSS and leader sequence analyses, position weight matrices (PWMs) were first generated for the gorilla and mouse datasets by parsing IMGT-derived annotations using the parse_imgt_annotations function. For each species and region, multiple annotation CSV files were merged, and PWMs were calculated using the calc_motifs function. Next, gapped V segment reference libraries were downloaded from the IMGT website using extract_refs. For the rhesus macaque, gaps in the reference sequences were corrected using the fix_macaque_gaps function to ensure compatibility with Digger’s input requirements. Digger was then executed on the same genomic regions as analyzed by VDJ-Insights, using identical gene segment libraries. These libraries were, however, first portioned into separate V, D, and J libraries for each species and region, as is required by Digger. All regions across all species were analyzed in both sense and antisense orientations (e.g. digger [region_fasta]—locus [locus]—species [species]—v_ref [V-library]—d_ref [D-library]—j_ref [J-library]—v_ref_gap [gapped-V-library]—sense [forwards/reverse] [output]). Next, the curated IMGT annotations were compared with the outcomes of VDJ-Insights and Digger using a custom script (available on GitHub). Annotations with overlapping or identical start and stop coordinates were considered concordant across IMGT, VDJ-Insights, and Digger.

### 2.4 Computational performance assessment

The computational efficiency of VDJ-Insights was evaluated by annotating a subset of 20 randomly selected human HPRC haplotypes using varying numbers of processing threads, ranging from 4 to 20. On a workstation equipped with 258 GB of RAM, VDJ-Insights completed IG and TCR region annotations within 20–40 min (Fig. 1A, available as supplementary data at *Bioinformatics* online). The tool operates with a minimum of 4 threads and achieves optimal performance when run with approximately 12 threads.

Regarding runtime, VDJ-Insights is computationally more efficient than Digger. A direct comparison is not entirely straightforward because Digger runs on a single processing thread, whereas VDJ-Insights requires at least four threads. Even so, on a workstation with 502 GB of RAM, Digger completed the full annotation in 7 h 18 min (SD = 0.01 h), while VDJ-Insights, using four threads on the same machine, completed the task in 1 h 35 min (SD = 0.02 h) (Fig. 1B, available as supplementary data at *Bioinformatics* online).

### 2.5 RSS and CDR feature visualization

The RSS and CDR regions were analyzed using two custom scripts. For the RSS analyses, only unique allele and corresponding RSS combinations of functional gene segments were used. Sequence motifs were generated for each region using the Python package Logomaker (v0.8.4) (Tareen and Kinney 2020).

For the CDR analyses, CDR1 and CDR2 regions were extracted from all unique alleles. The corresponding nucleotide sequences were translated to amino acids sequences, which were then aligned using Clustal-Omega (v1.2.4) (Sievers *et al.* 2011). Subsequently, sequence motifs were generated from the aligned amino acid sequences using Logomaker (Tareen and Kinney 2020).

## 3 Results

### 3.1 Benchmarking VDJ-Insights using IMGT-curated IG and TCR regions from human, nonhuman primates, and mice

To validate the accuracy and robustness of VDJ-Insights, we applied the tool to annotate IMGT-curated IG and TCR regions from a human, four gorillas, a rhesus macaque, and four house mouse strains (Table 2, available as supplementary data at *Bioinformatics* online) (Guo *et al.* 2017, Tran et al. 2021, Nguefack Ngoune *et al.* 2022, Debbagh *et al.* 2024). For each species, the latest gene segment libraries, comprising functional, open-reading frame (ORF), and pseudogene segments, were sourced from the IMGT database (release 30 April 2025) (Lefranc 2011). This setup was also applied to run Digger, a recently published IG and TCR annotation tool, allowing a direct comparison of performance (Lees *et al.* 2024).

**Figure 2:**
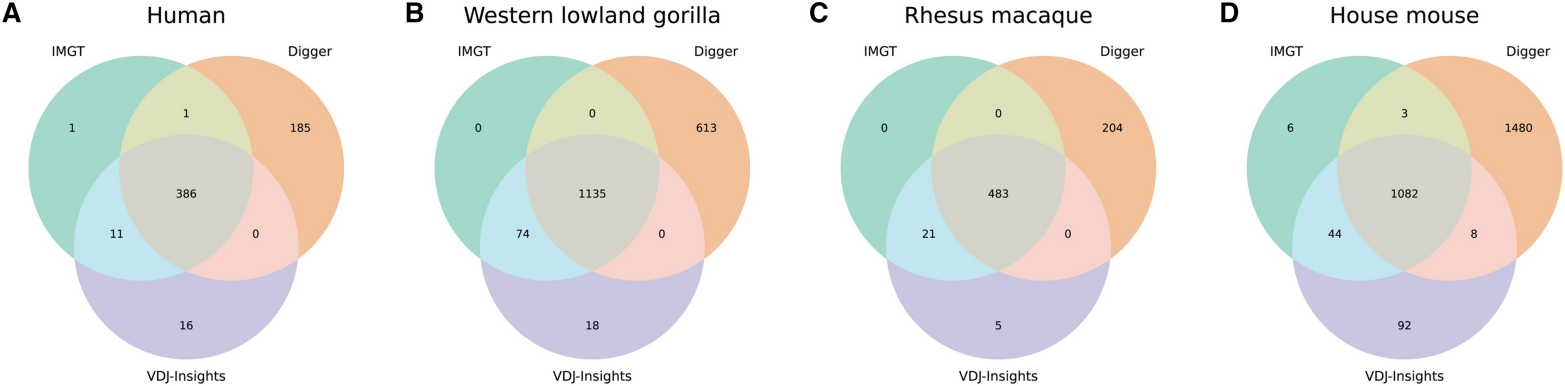
Comparison of gene segment annotations by VDJ-Insights, Digger, and IMGT. Venn diagrams depicting the overlap of annotated V, D, and J gene segments at both IG and TCR loci as identified by VDJ-Insights, Digger, and IMGT across the curated assemblies of a human (A), four gorillas (B), a rhesus macaque (C), and four mouse strains (D).

The curated human dataset includes the IGH and all TCR loci, comprising a total of 399 gene segments according to IMGT annotation. Using VDJ-Insights, we correctly identified all but two gene segments (Fig. 2A). Additionally, the tool reported 16 extra gene segments absent from the IMGT annotation, which may represent false positives or gene segments not yet annotated by IMGT. These extra gene segments were predominantly D segments, which are inherently challenging to map accurately due to their short length, along with two V segments that were identical to known reference sequences. In comparison, Digger identified 387 of the 399 IMGT-curated segments and reported 185 additional gene segments, which may represent false positives, including 79 V segments (Fig. 2A). VDJ-Insights was subsequently applied to curated IG loci from gorilla, rhesus macaque, and mouse (IGH and IGL), where it also achieved higher annotation concordance than Digger in all three species (Fig. 2B–D; Table 3, available as supplementary data at *Bioinformatics* online).

**Figure 3:**
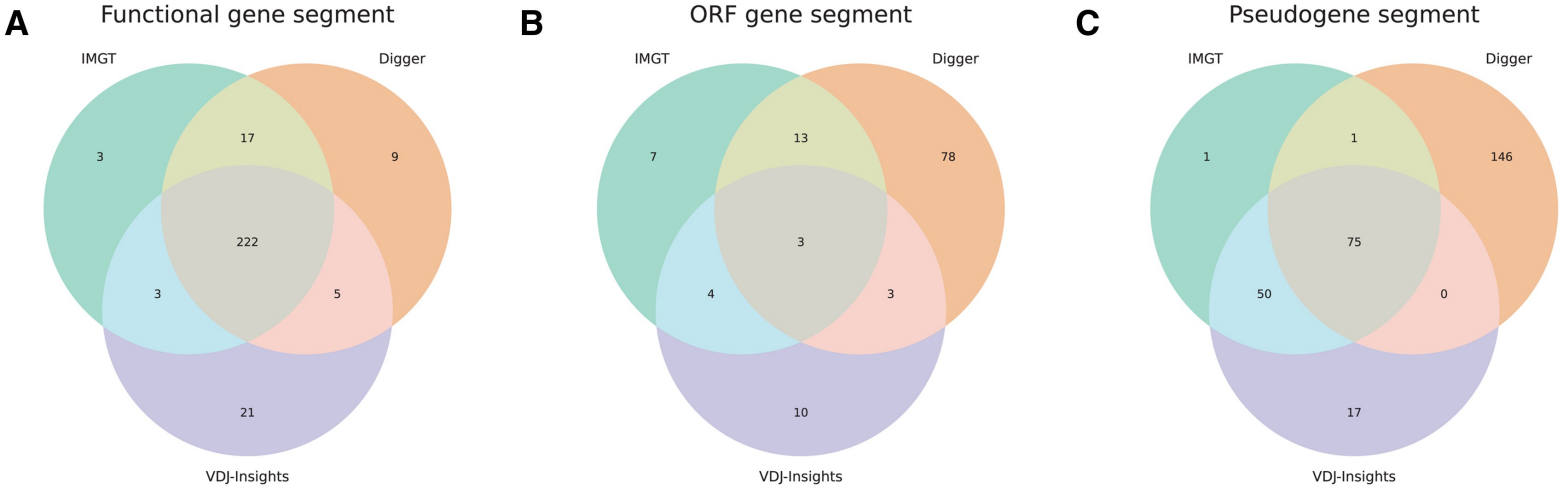
Comparison of functionality classification by VDJ-Insights, Digger, and IMGT. Venn diagrams illustrating the overlap in functionality classifications assigned by VDJ-Insights and Digger, compared to IMGT-curated classifications, for human gene segments categorized as functional (A), open-reading frame (ORF) (B), and pseudogenes (C).

Next, we evaluated the accuracy of gene functionality classification as predicted by VDJ-Insights. The tool annotated 251 human gene segments as functional, 20 as ORFs, and 142 as pseudogenes, corresponding to concordance rates of 91.8%, 25,9%, and 98.4% with IMGT-curated classifications, respectively (Fig. 3; Table 4, available as supplementary data at *Bioinformatics* online). The observed discrepancies predominantly involved segments designated by IMGT as functional or ORFs (Table 5, available as supplementary data at *Bioinformatics* online), potentially reflecting the incorporation of additional information, such as experimental transcriptomic data, in the IMGT curation process (Giudicelli and Lefranc 2012). As compared to VDJ-Insights, Digger showed higher concordance with IMGT-curated functionality classifications for functional and ORF segments in humans, achieving agreement rates of 97.6% and 59.3%, respectively (Fig. 3; Table 4, available as supplementary data at *Bioinformatics* online). However, its agreement rate in classifying pseudogene segments was lower, with a concordance rate of 59.8%. The higher concordance of Digger for functional and ORF classifications is likely a consequence of its default integration of IMGT-derived matrices, which incorporate curated information on gene functionality. A key limitation of this approach, however, is that Digger can only annotate in species for which such matrices are available. In contrast, the annotation process of VDJ-Insights does not depend on predefined references and is therefore applicable to a broader range of species (Lees *et al.* 2024). In the gorilla, rhesus macaque, and mouse datasets, functional and ORF classifications were largely consistent between the two tools, with Digger achieving higher concordance on functionality and ORF classification, whereas VDJ-Insights provided a more accurate annotation of pseudogene segments (Fig. 2 and Tables 6–8, available as supplementary data at *Bioinformatics* online).

Overall, we validated the accuracy and robustness of VDJ-Insights in identifying gene segments and classifying their functionality with high concordance to curated references. The reference-free approach positions VDJ-Insights as a valuable tool for extending IG and TCR region analyses beyond model species with well-curated gene libraries.

### 3.2 Characterization of IG and TCR regions in the human pangenome dataset

The performance and computational efficiency of VDJ-Insights on large-scale datasets was evaluated by characterizing the IG and TCR regions across 47 phased diploid human assemblies obtained from the HPRC and the T2T reference genome of the CHM13 cell line (Nurk *et al.* 2022, Liao *et al.* 2023).

Although VDJ-Insights successfully identified the flanking genes in all samples, not all IG and TCR regions appeared to be completely or correctly assembled (Fig. 3, available as supplementary data at *Bioinformatics* online). This was most evident at the IGH and IGK loci, where only three and four regions, respectively, were presumed complete. These assembly inconsistencies likely originate from the LCL origin of the HPRC samples, in which somatic rearrangements reduce representation of germline haplotypes (Rodriguez *et al*. 2021, Ford *et al.* 2025). Another contributing factor is the intrinsic complexity of IG and TCR regions, which are repeat-rich sequences. For IGH and IGK in particular, their proximity to centromeric and telomeric regions of the chromosomes further complicates assembly and can result in fragmentation across multiple contigs (McBride *et al.* 1982, Lefranc and Lefranc 2001, Liao *et al.* 2023, Zhu *et al.* 2025). VDJ-Insights addresses region fragmentation through an optional scaffolding step in the pipeline that uses a T2T genome as a reference to stitch fragmented contigs into a more contiguous scaffold (Nurk *et al.* 2022). This approach enabled the recovery of an additional 26 IG loci (Fig. 3, available as supplementary data at *Bioinformatics* online). Scaffolded regions, consisting of multiple contigs, are flagged in the annotation report, as these assemblies may not fully capture their complete gene segment content. In total, 392 regions from the HPRC and T2T datasets were successfully annotated, including 115 IG (IGH, IGK, and IGL) and 277 TCR (TRA–TRD, TRB, and TRG) regions.

Two complementary public databases are available for human V, D, and J gene segments: IMGT, comprising curated germline sequences, and VDJbase, which contains genomic sequences from over 200 individuals (Lefranc 2011, Omer *et al.* 2020). Although VDJ-Insights is configured to access the most recent IMGT gene segment library by default, users can optionally utilize custom libraries. For the annotation of IG regions in the HPRC datasets, we employed three gene segment libraries: IMGT, VDJbase, and a custom combined library. The IMGT library contained 1051 unique IG alleles (release 30 April 2025), while the VDJbase library comprises 980 unique IG alleles (assessed 30 April 2025), with 347 alleles shared between these libraries. A combined library was generated by retaining one representative for each shared IG sequence and including all library-specific sequences, resulting in a total of 1684 unique alleles.

Using the IMGT library, a total of 26 865 gene segments were annotated across the IGH, IGK, and IGL loci from the 94 HPRC haplotypes and the T2T genome. In comparison, the VDJbase library annotated 13 779 gene segments. Utilizing the combined library, VDJ-Insights annotated a total of 27 514 gene segments (Fig. 4, available as supplementary data at *Bioinformatics* online; Table 2). Among these annotated gene segments, 1428 were unique alleles, of which 652 were novel. Of these novel segments, 118 were confirmed in at least four individuals, providing strong evidence for their authenticity. Notably, many novel J-segments were detected, but only two were identified in at least four individuals, which likely reflects the LCL origin of the samples. Cross-comparison of the different libraries showed that 13 123 gene segments were consistently identified by all three reference datasets, whereas 13 742 and 656 gene segments were exclusively annotated by the IMGT and VDJbase libraries, respectively. These findings highlight the importance of comprehensive reference libraries for enhancing the accuracy of gene segment annotation.

**Table 2:** Alleles annotated across the HPRC cohort for both the IG and TCR regions.

	IGV	IGD	IGJ	Total	TRV	TRD	TRJ	Total
Alleles identified by								
IMGT library	24 667	866	1332	26 865	13 642	1496	7882	23 020
VDJbase library	11 037	959	1783	13 779				
Combined library	24 662	959	1893	27 514				
Alleles annotated								
Unique alleles	1274	40	114	1428	484	6	117	607
Unique novel alleles	561	10	81	652	250	0	25	275
Novel alleles present in at least two individuals	199	2	4	205	125	0	12	137
Novel alleles present in at least four individuals	114	2	2	118	88	0	5	93

Since VDJbase exclusively includes V, D, and J gene segments for IG loci, TCR region annotation was performed solely using the IMGT library. In total, 23 020 TCR gene segments were annotated in the 95 human haplotypes, representing 607 unique alleles (Table 2). Of these unique alleles, 275 were identified as novel compared to the current IMGT reference. Furthermore, among these novel segments, 137 were documented in multiple individuals, providing additional support for their validity. At a haplotype level, we detected identical TCRB and TCRG haplotypes among HPRC samples, although at low frequencies (Fig. 5E and F, available as supplementary data at *Bioinformatics* online). These identical haplotypes were mainly identified in individuals of the same ethnicity (Liao *et al.* 2023), suggesting the presence of conserved TCR configurations within specific populations. All 46 studied individuals were heterozygous at their IG and TCR loci, except for 1 individual (HG01928, haplotypes GCA_018472695.1 and GCA_018472705.1), who was homozygous for the TCRG locus.

In addition to gene segment annotation, VDJ-Insights automatically identifies the RSS, as well as the CDR1 and CDR2 for the annotated IG and TCR gene segments. To assess nucleotide variation, sequence motifs were generated for each of these features across all IG and TCR loci. Analysis of the RSS of functional gene segments revealed highly conserved motifs across loci (Fig. 6, available as supplementary data at *Bioinformatics* online), consistent with the conserved nature of the RAG1 and RAG2 recombinase proteins that mediate V(D)J recombination (Liu *et al.* 2022). An exception to these conserved motives was observed in the TRDD segments, likely due to annotation challenges associated with their short segment length. This may result in false positives that obscure true motif patterns. Other loci showed position-specific variability within otherwise conserved motifs. For example, the fourth position of the V-nonamer sequence displayed greater variability across all three IG loci, suggesting that this position may play a less critical role in RAG complex binding. As expected, the CDR regions exhibit substantial sequence variation (Fig. 7, available as supplementary data at *Bioinformatics* online), consistent with their central role in forming the antigen-binding sites and binding of IG and TCR receptors to the major histocompatibility complex (MHC) (Wong *et al.* 2019). The CDR sequences were identified using IMGT-derived reference coordinates, resulting in the annotation of 757 sequences for IG loci and 390 sequences for TCR loci. Although not all gene segments in the IMGT database include annotated CDR regions, the majority of functional and ORF-classified V segments do contain this information. In contrast, limited CDR sequences are documented for the TRD and TRG loci, largely due to their relatively small number of V gene segments. Sequence motif analysis of the CDRs displayed extensive variability, with no universally conserved amino acid positions. Nevertheless, certain residues appeared with increased frequency at specific positions. For example, IGH-CDR1 frequently begins with glycine, while TRBV-CDR1 often includes a histidine. Additionally, the length of CDRs varied extensively, exemplified by IGHV-CDR2, which ranged from 3 to 10 amino acids in length. This high degree of diversity in both sequence composition and length highlights the evolutionary importance of CDR variability in supporting the broad antigen recognition capabilities of the adaptive immune system.

Overall, the in-depth characterization of IG and TCR immune regions in the HPRC cohort highlights the strength and robustness of our annotation tool. With the growing availability of large-scale genomic data, VDJ-Insights provides a comprehensive and benchmarked solution for automated IG and TCR annotation, advancing our understanding of the germline diversity that underlies adaptive immune responses.

## 4 Discussion

Despite the central role of IG and TCR gene regions in the adaptive immune response, comprehensive germline characterization of these loci has historically remained limited due to their complexity (Kidd *et al.* 2012). Recent advances in long-read and high-throughput sequencing technologies have substantially improved the potential to characterize these regions (Rodriguez *et al.* 2020, Rodriguez *et al.* 2022, Gibson *et al.* 2023, Rodriguez *et al.* 2023, Engelbrecht *et al.* 2024). However, the accurate identification and annotation of IG and TCR gene segments remains a challenging and time-intensive task, necessitating robust analytical tools and expert curation. VDJ-Insights was developed to address this challenge, providing a fast and reliable platform capable of annotating both known and novel gene segments across the highly polymorphic IG and TCR regions.

When benchmarked against expert-curated datasets from a human, gorillas, a rhesus macaque, and mice, VDJ-Insights successfully identified over 99% of the annotated segments (Table 3, available as supplementary data at *Bioinformatics* online). VDJ-Insights also identified several additional gene segments absent from the curated references. Many of these were D gene segments, which are particularly difficult to annotate due to their short length, sometimes as small as eight base pairs. For the few additional V segments, further investigation is warranted to determine whether these represent genuine new gene segment annotations or potential false positives. It may also be possible that some curated references are incomplete and therefore miss certain annotations. VDJ-Insights showed strong concordance in functionality classification with the reference annotations (Table 4, available as supplementary data at *Bioinformatics* online). Discrepancies in our predicted functionality classification mostly involved segments labeled as ORFs. Several factors may account for these differences. For instance, while IMGT assigns a single functionality status to each allele, we can now demonstrate that variations in RSS or leader sequences can occur among identical allele sequences, potentially resulting in distinct functionality outcomes for the same allele. Moreover, IMGT’s integration of transcriptomic datasets, where available, further informs their functional assignments. As a result, functionality classification of annotation tools like VDJ-Insights, which rely exclusively on genomic sequence data and predefined rules, may occasionally diverge from IMGT’s more nuanced classifications. Functionality classifications based solely on genomic sequences should be regarded as provisional and meant to provide a first indication rather than a confirmed classification.

During validation, we compared VDJ-Insights with Digger, a recently developed tool for IG and TCR gene annotation, which demonstrated slightly higher performance in predicting functional gene segments (Lees *et al.* 2024). This enhanced performance is likely due to Digger’s use of PWMs derived from IMGT-curated annotations. While this approach increases functional classification accuracy for well-characterized species, it limits the applicability of Digger to species or alleles represented in existing reference datasets. In contrast, VDJ-Insights is designed to minimize dependence on external reference databases. This makes VDJ-Insights particularly well suited for application to understudied or nonmodel organisms, including those lacking comprehensive IG or TCR gene annotations. Furthermore, VDJ-Insights is capable of processing multiple samples in parallel, enabling high-throughput analysis for large-scale genomic studies, such as pangenome assemblies and population-level cohorts. We demonstrated this scalability by annotating 94 haplotypes from the HPRC and the T2T human reference genome (Nurk *et al.* 2022, Liao *et al.* 2023), highlighting the efficiency and robustness of VDJ-Insights across extensive datasets.

The accuracy of annotations remains highly dependent on the quality of the underlying genome assemblies. This is especially true for the IGH, IGK, and IGL regions in the HPRC assemblies derived from LCLs. Some monoclonal LCLs can include somatic deletions of up to 940 kb in the IGH region (Rodriguez *et al.* 2021). Our analyses suggest that such rearrangements and somatic deletions most often affect IGH and IGK, which likely explains the high prevalence of incomplete and fragmented regions. This observation aligns with the order of rearrangements during B cell maturation (Hieter *et al.* 1981).

Although HPRC assemblies are of high quality, generated using a combination of Pacific Biosciences HiFi reads, Oxford Nanopore long reads, Bionano optical maps, and Hi-C Illumina short reads, several IG regions remained fragmented and incomplete across multiple contigs (Liao *et al.* 2023). To address these challenges, VDJ-Insights provides the option to implement a reference-guided scaffolding strategy to improve contiguity of fragmented regions. This extra feature enhances the capacity to produce accurate and more complete annotations, even in fragmented assemblies.

In addition to the need for high-quality assemblies, our analyses emphasize the critical role of a comprehensive and well-curated gene segment library in achieving accurate IG and TCR annotations. This became evident during comparative evaluations using different reference libraries. Notably, when relying exclusively on the VDJbase library, a substantial number of gene segments were not annotated compared to results obtained using the IMGT or combined library (Omer *et al.* 2020). Utilizing larger and more inclusive gene segment libraries may not only improve detection sensitivity, but also enhances the accuracy of functionality predictions. This is due to the adaptive modeling of RSS motifs using PWMs. As VDJ-Insights dynamically adjusts motif models based on the reference segments provided, richer libraries enable more robust functionality assessments. Although larger gene segment libraries increased the number of annotations, the quality of those libraries is as important for accurate IG and TCR annotation (Collins *et al.* 2025). This highlights the need for quality-control guidelines and transparency in publicly available datasets (Watson *et al.* 2025).

VDJ-Insights accommodates postanalysis updates to reference libraries, allowing reanalysis with newly added or revised gene segments. This feature facilitates the annotation of previously unrecognized or misclassified segments, particularly as more novel alleles are discovered through large-scale sequencing efforts. However, it is essential to carefully evaluate the quality and reliability of added gene segments, especially for short segments such as D genes, which are inherently more prone to false-positive matches due to their limited sequence complexity.

Our analysis of the IG and TCR loci across the HPRC cohort resulted in the annotation of 2035 unique alleles, of which 927 represent novel sequences not previously cataloged in IMGT or VDJbase. Among these novel segments, 211 were identified in at least four individuals, supporting their validity (Table 2). RSS motifs were extracted from all annotated segments and revealed a high degree of sequence conservation, consistent with their essential role in guiding V(D)J recombination. In parallel, CDR1 and CDR2 regions were annotated for 1070 alleles using reference coordinates derived from IMGT. As expected, these regions displayed substantial sequence variability, which underlies their importance in directly recognizing antigens and engaging peptides presented by MHC molecules (Wong *et al.* 2019). Both RSS and CDR annotations offer valuable opportunities for downstream analyses. For example, variations in RSS motifs, in conjunction with segment positioning within the regions, have been linked to differences in V(D)J recombination frequency (Williams *et al.* 2001, Wu *et al.* 2003). Similarly, availability of genomic CDR sequences enables investigations into somatic hypermutation and the functional evolution of the immune repertoire (Wong *et al.* 2019). These features extend the utility of VDJ-Insights beyond gene segment annotation, offering a framework for functional immunogenetic as well as evolutionary comparative exploration at the individual, population, and species levels.

## Supplementary Material

btag108_Supplementary_Data

## Data Availability

Datasets and software package underlying this article are available in the VDJ-insights repository, https://github.com/BPRC-Bioinfo and https://doi.org/10.5281/zenodo.17588835. Additional intermediate datasets used and analysed during the current study are available from the corresponding authors upon reasonable request.
