# Supplementary material for: VDJ-Insights: simplifying the annotation of genomic immunoglobulin and T cell receptor regions
Source: Bioinformatics. 2026 Mar 9;42(4):btag108. doi: 10.1093/bioinformatics/btag108 (PMC13064985; doi:10.1093/bioinformatics/btag108)
Supplement: btag108_Supplementary_Data [file btag108_supplementary_data.zip › Supplemental-figures-Ott-et-al.pdf]

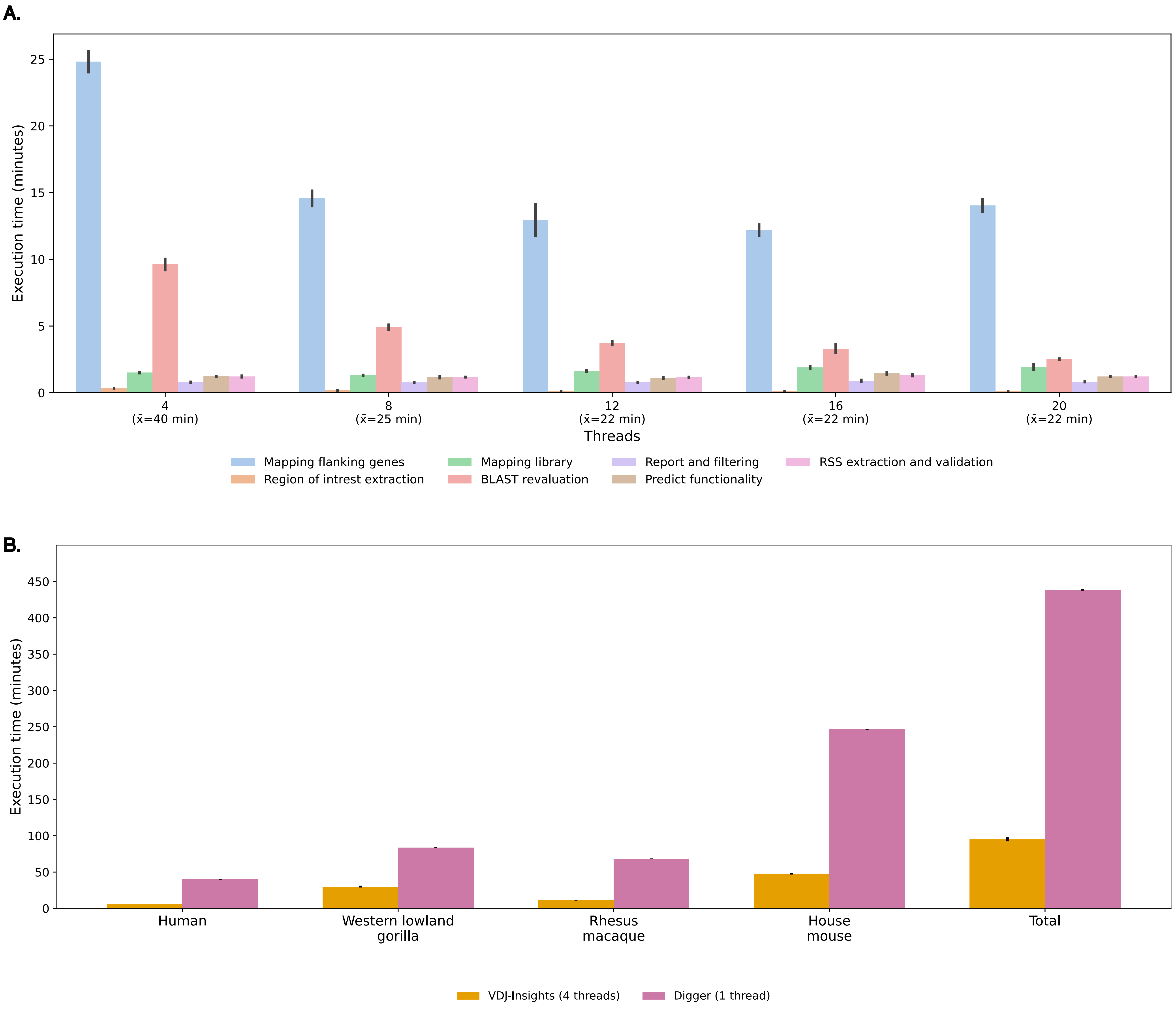

Supplemental figure S1. Execution times and computational benchmarking of VDJ-Insights. (A) Execution times of individual pipeline steps of VDJ-Insights. The TCR regions from 20 randomly selected samples from the Human Pan-Genome Reference Consortium (HPRC) were extracted and annotated using their whole-genome assemblies. The analysis was performed on an AMD EPYC 7F72 24-Core Processor (utilizing 12 cores, 24 threads) with 258 GB RAM. Each colour corresponds to a distinct step in the VDJ-Insights pipeline, as detailed in the pipeline flowchart (Fig. 1). The x-axis indicates the number of threads utilized (4, 8, 12, 16, and 20). Results shown are averages (x·) across three independent runs, with error bars representing the standard deviation (SD). (B) Runtime comparison between VDJ-Insights and Digger. Analyses were performed on an AMD EPYC 7F72 24-core processor with 502 GB RAM. Digger operates exclusively in single-threaded mode, whereas VDJ-Insights requires a minimum of four threads, resulting in different benchmarking conditions. Time is reported in minutes per species and for the total analysis, with error bars representing the SD in minutes.

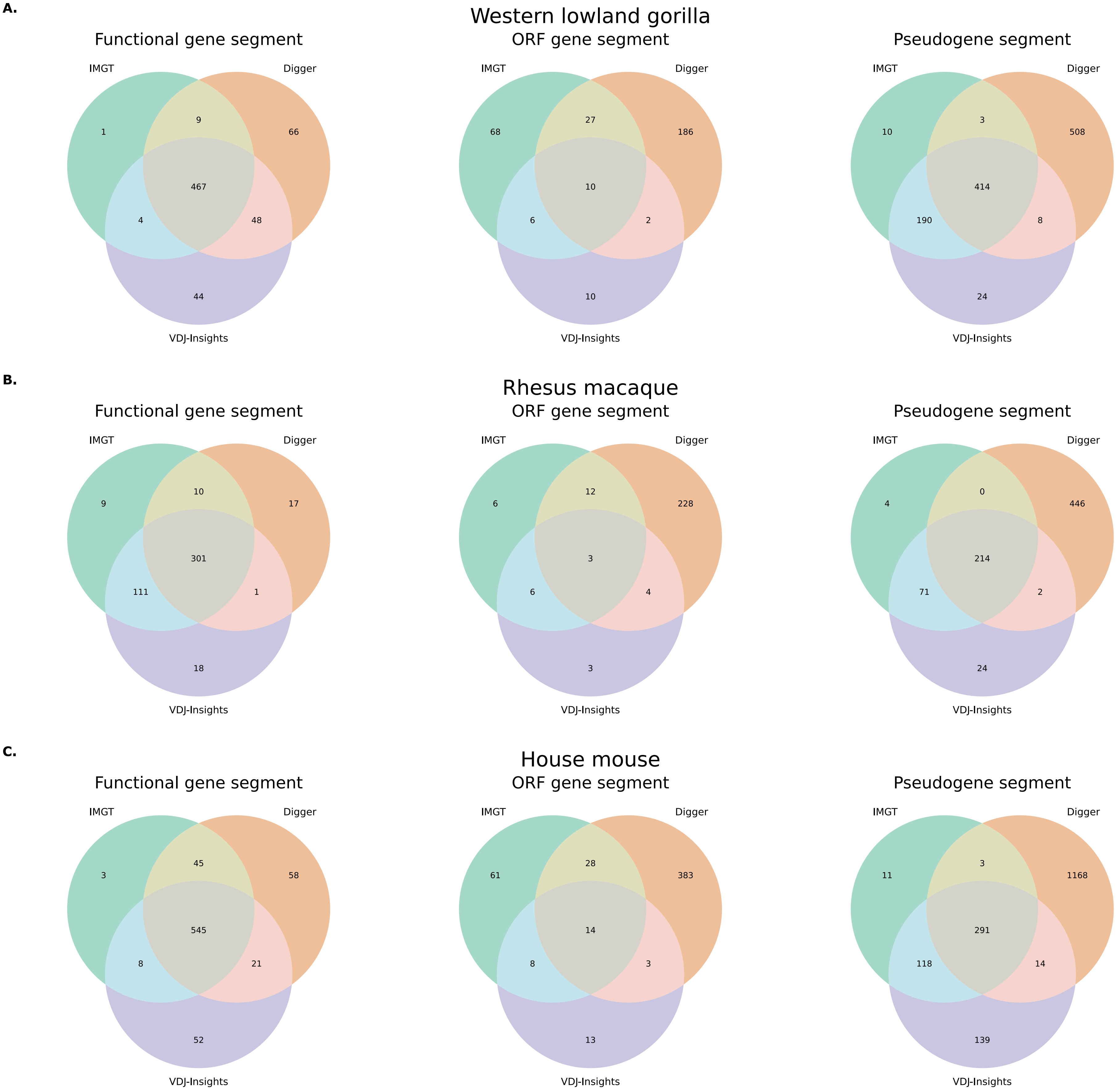

Supplemental figure S2. Comparison of functionality classification by VDJ-Insights, Digger, and IMGT. Venn diagrams illustrating the overlap in functionality classifications assigned by VDJ-Insights and Digger, compared to IMGT curated classifications, for gorillas (A), the rhesus macaque (B), and mice (C). Gene segments are categorized as functional, open read-ing frame (ORF), and pseudogenes.

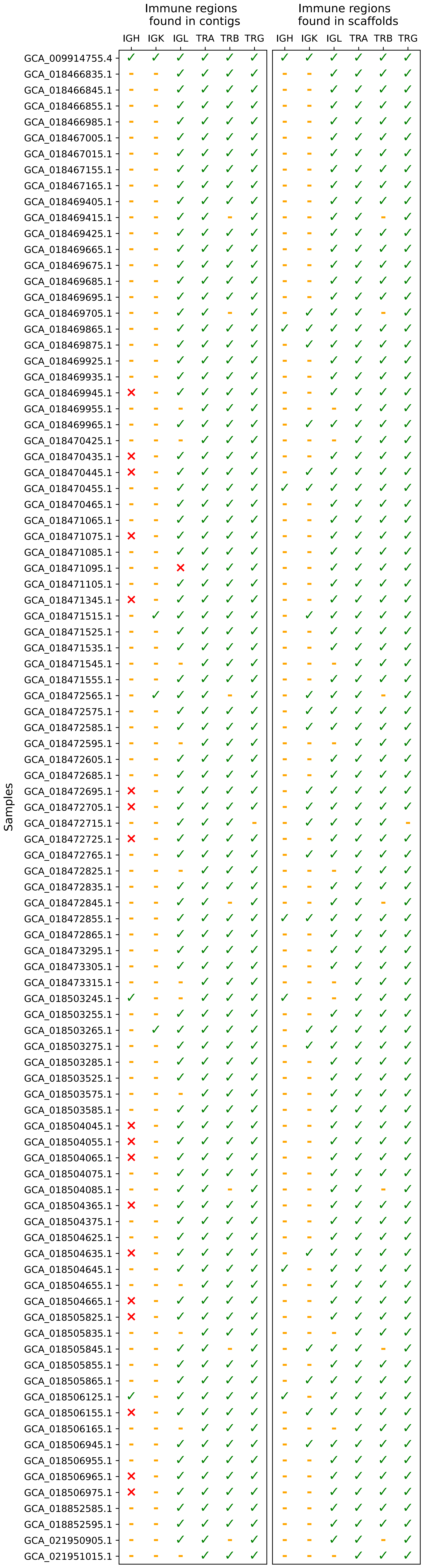

Supplemental figure S3. Overview of IG and TCR regions identified across the HPRC cohort. IG and TCR genomic regions were extracted and annotated from the whole-genome sequence contigs of the HPRC cohort (release 1, left) and the VDJ-Insights-scaffolded assemblies (right). The annotation status for each genomic region is depicted. A green check mark indicates successful extraction and annotation, defined as the locus being assembled onto a single contig or scaffold, with the number of annotated V, D, and J gene segments falling within the expected range as specified by IMGT (46, 61). An orange dash signifies an incomplete annotation, typically due to locus fragmentation across multiple contigs or annotation of fewer than 20% of the expected gene segments. A red cross denotes complete absence of annotated gene segments in the given region.

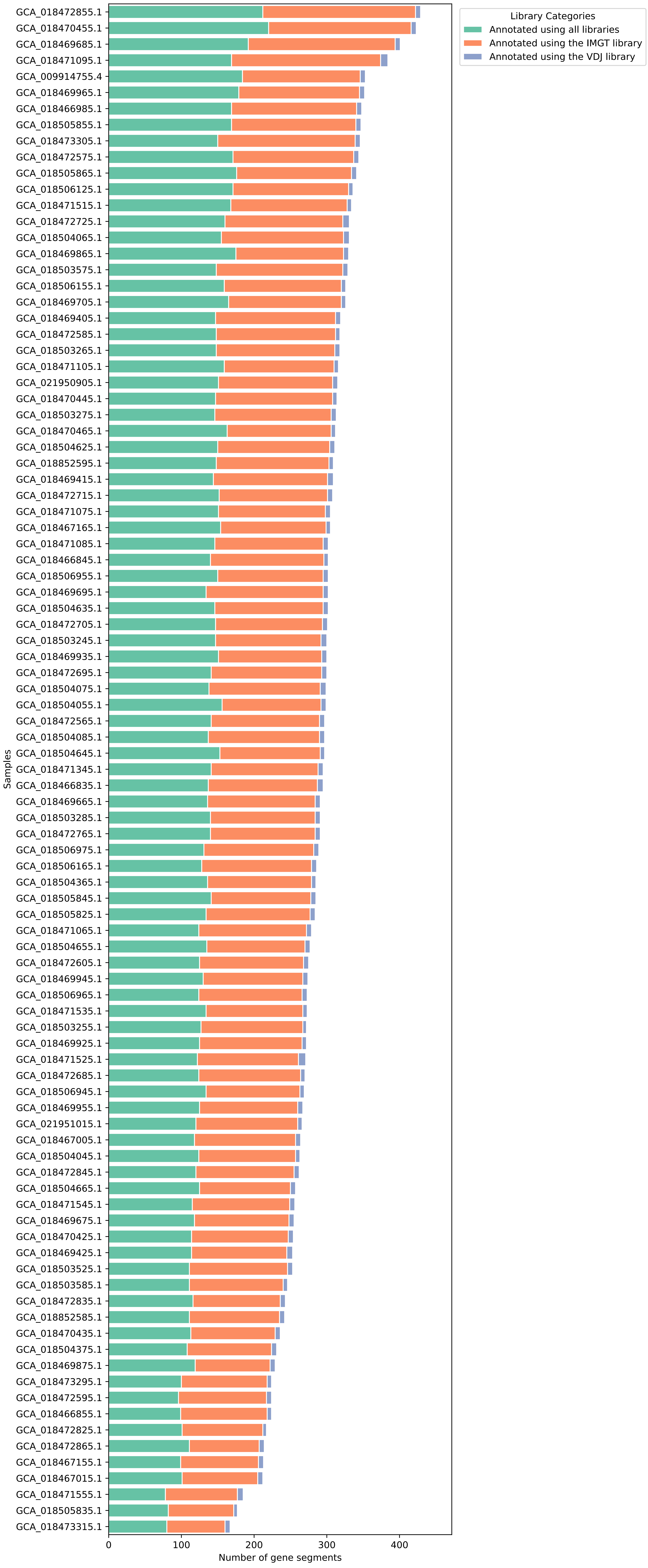

Supplemental figure S4. Number of gene segments annotated using different reference libraries. The bar chart illustrates how many gene segments were identified per haplotype, with colours indicating detection by the IMGT li-brary, VDJbase library, or a combined library.

Supplemental figure S5. Visualization of gene segment presence across haplotypes in different IG and TCR regions. Each subplot (A-F) shows a different IG or TCR region. The boxes indicate the presence (coloured) or absence (blank) of a specific gene segment per haplotype. Colours distin-guish known alleles from novel ones based on existing gene segment li-braries. Numbers within the boxes represent the number of duplicated seg-ments. If multiple duplications are present, intermediate shading indicates the proportion of known versus novel alleles. The side chart illustrates the presence of gene segment families per haplotype, and the top chart summa-rizes the number of segments classified as functional, ORF or non-functional by VDJ-Insights.

A.

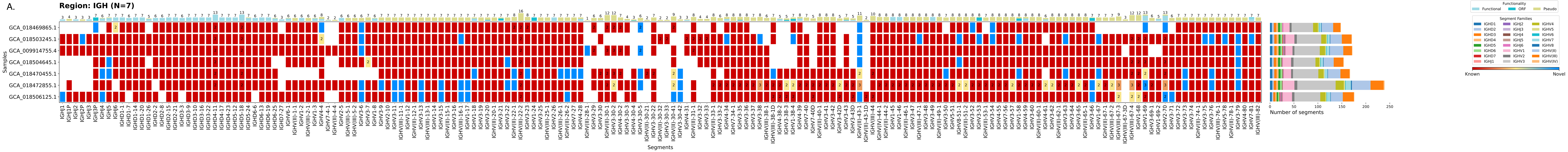

B.

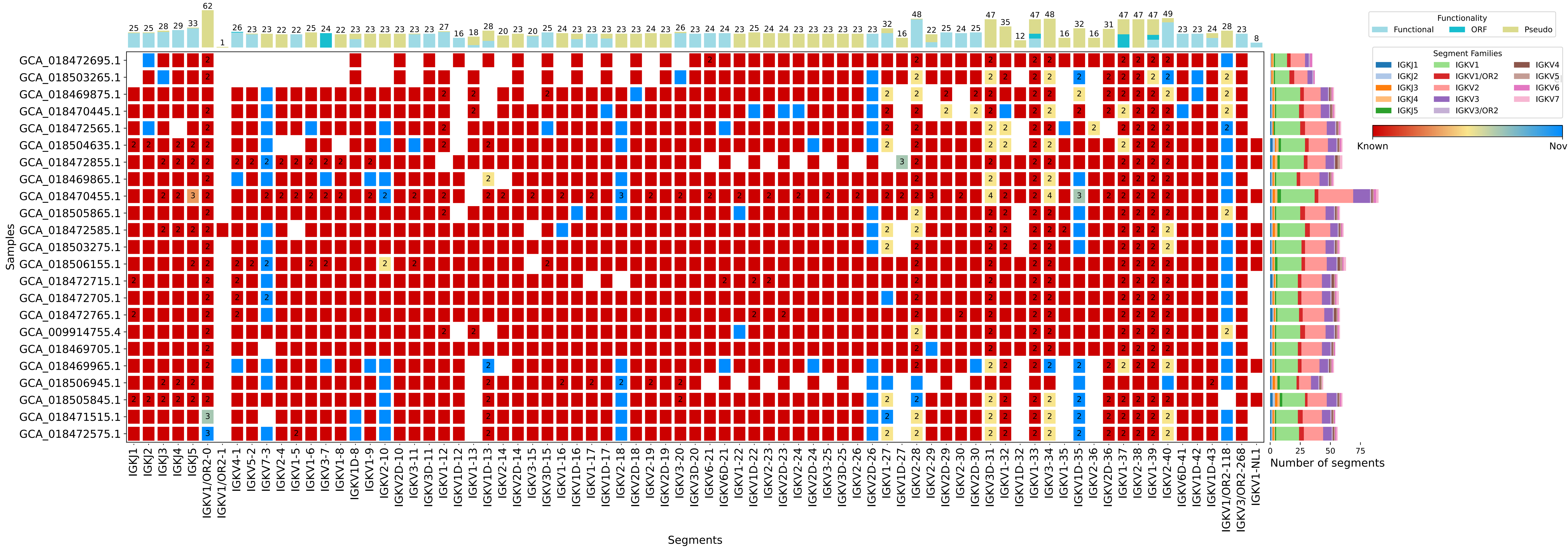
Region: IGK (N=23)
Functionality
Functional
ORF
Pseudo
Segment Families
IGKJ1
IGKJ2
IGKJ3
IGKJ4
IGKJ5
IGKV1
IGKV1/OR2
IGKV2
IGKV3
IGKV3/OR2
IGKV4
IGKV5
IGKV6
IGKV7
Known
Nov
Samples
GCA_018472695.1
GCA_018503265.1
GCA_018469875.1
GCA_018470445.1
GCA_018472565.1
GCA_018504635.1
GCA_018472855.1
GCA_018469865.1
GCA_018470455.1
GCA_018505865.1
GCA_018472585.1
GCA_018503275.1
GCA_018506155.1
GCA_018472715.1
GCA_018472705.1
GCA_018472765.1
GCA_009914755.4
GCA_018469705.1
GCA_018469965.1
GCA_018506945.1
GCA_018505845.1
GCA_018471515.1
GCA_018472575.1
Segments
IGKJ1
IGKJ2
IGKJ3
IGKJ4
IGKJ5
IGKV1/OR2-0
IGKV1/OR2-1
IGKV4-1
IGKV5-2
IGKV7-3
IGKV2-4
IGKV1-5
IGKV1-6
IGKV3-7
IGKV1-8
IGKV1D-8
IGKV1-9
IGKV2-10
IGKV2D-10
IGKV3-11
IGKV3D-11
IGKV1-12
IGKV1D-12
IGKV1-13
IGKV1D-13
IGKV2-14
IGKV2D-14
IGKV3-15
IGKV3D-15
IGKV1-16
IGKV1D-16
IGKV1-17
IGKV1D-17
IGKV2-18
IGKV2D-18
IGKV2-19
IGKV2D-19
IGKV3-20
IGKV3D-20
IGKV6-21
IGKV6D-21
IGKV1-22
IGKV1D-22
IGKV2-23
IGKV2D-23
IGKV2-24
IGKV2D-24
IGKV3-25
IGKV3D-25
IGKV2-26
IGKV2D-26
IGKV1-27
IGKV1D-27
IGKV2-28
IGKV2D-28
IGKV2-29
IGKV2D-29
IGKV2-30
IGKV2D-30
IGKV3-31
IGKV1-32
IGKV1D-32
IGKV1-33
IGKV3-34
IGKV1-35
IGKV1D-35
IGKV2-36
IGKV2D-36
IGKV1-37
IGKV2-38
IGKV1-39
IGKV2-40
IGKV6D-41
IGKV1D-42
IGKV1D-43
IGKV1/OR2-118
IGKV3/OR2-268
IGKV1-NL1
0
25
50
75
Number of segments

C.

Region: IGL (N=85)

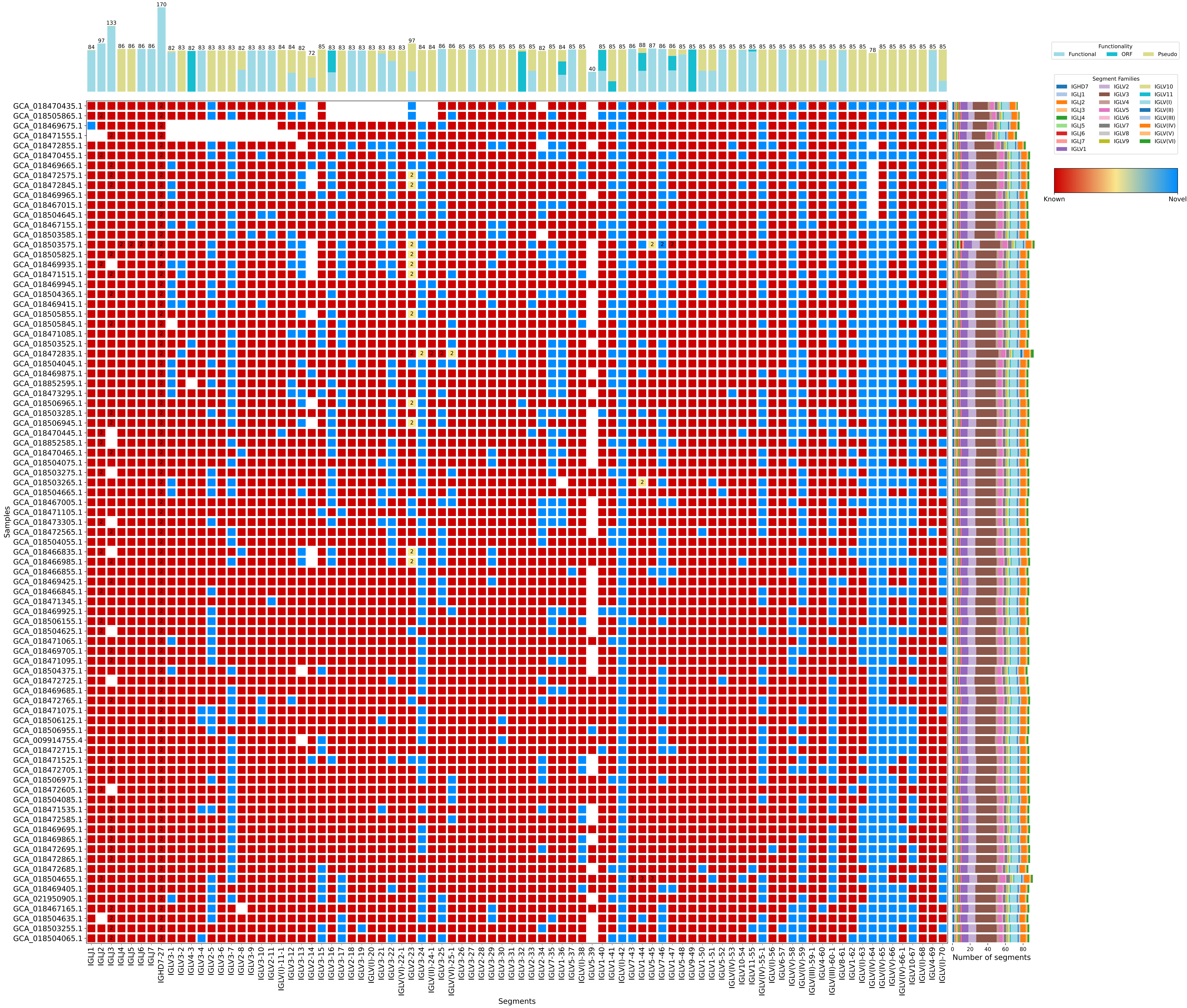

D.

Region: TRA (N=95)

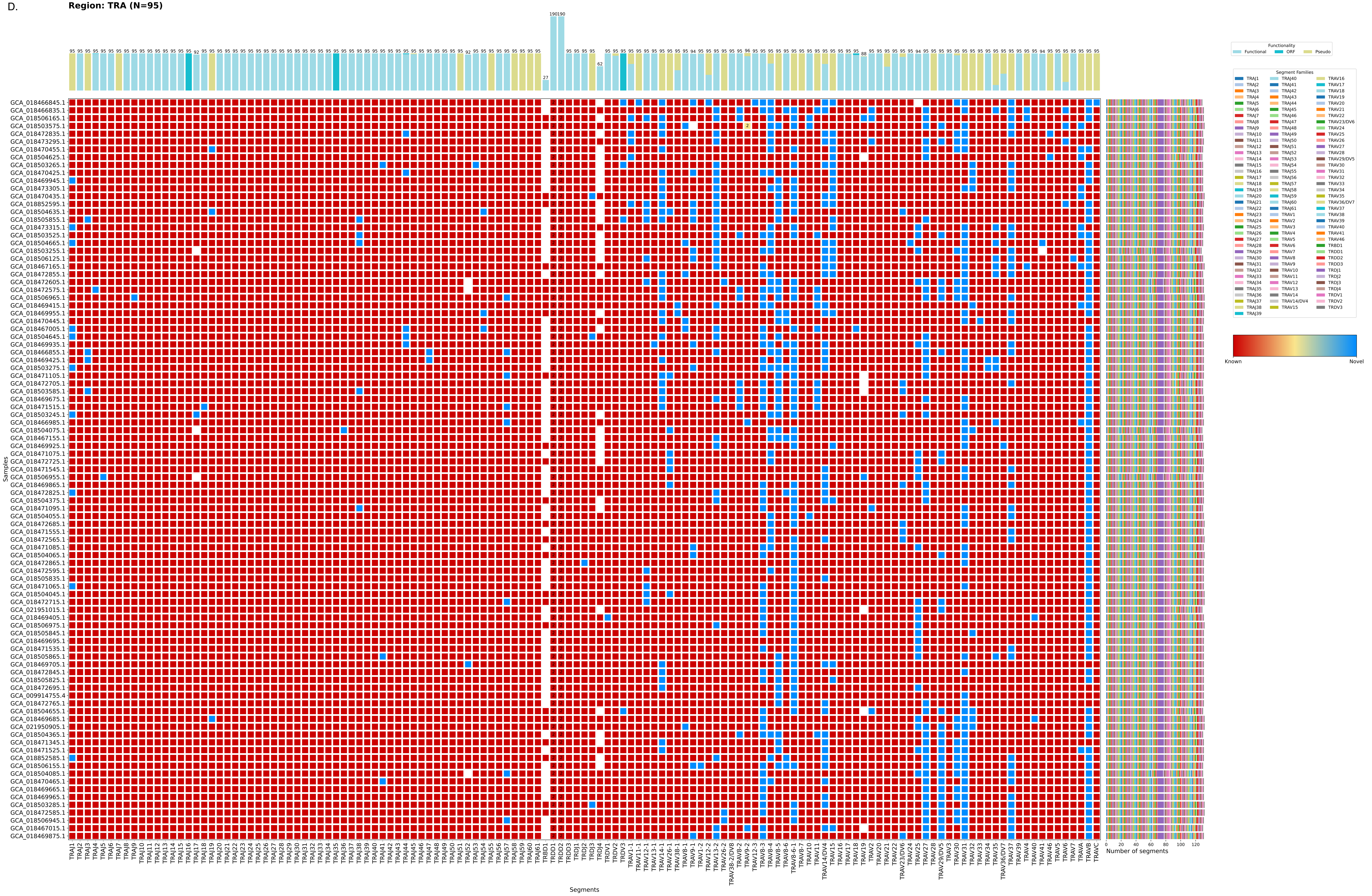

E.

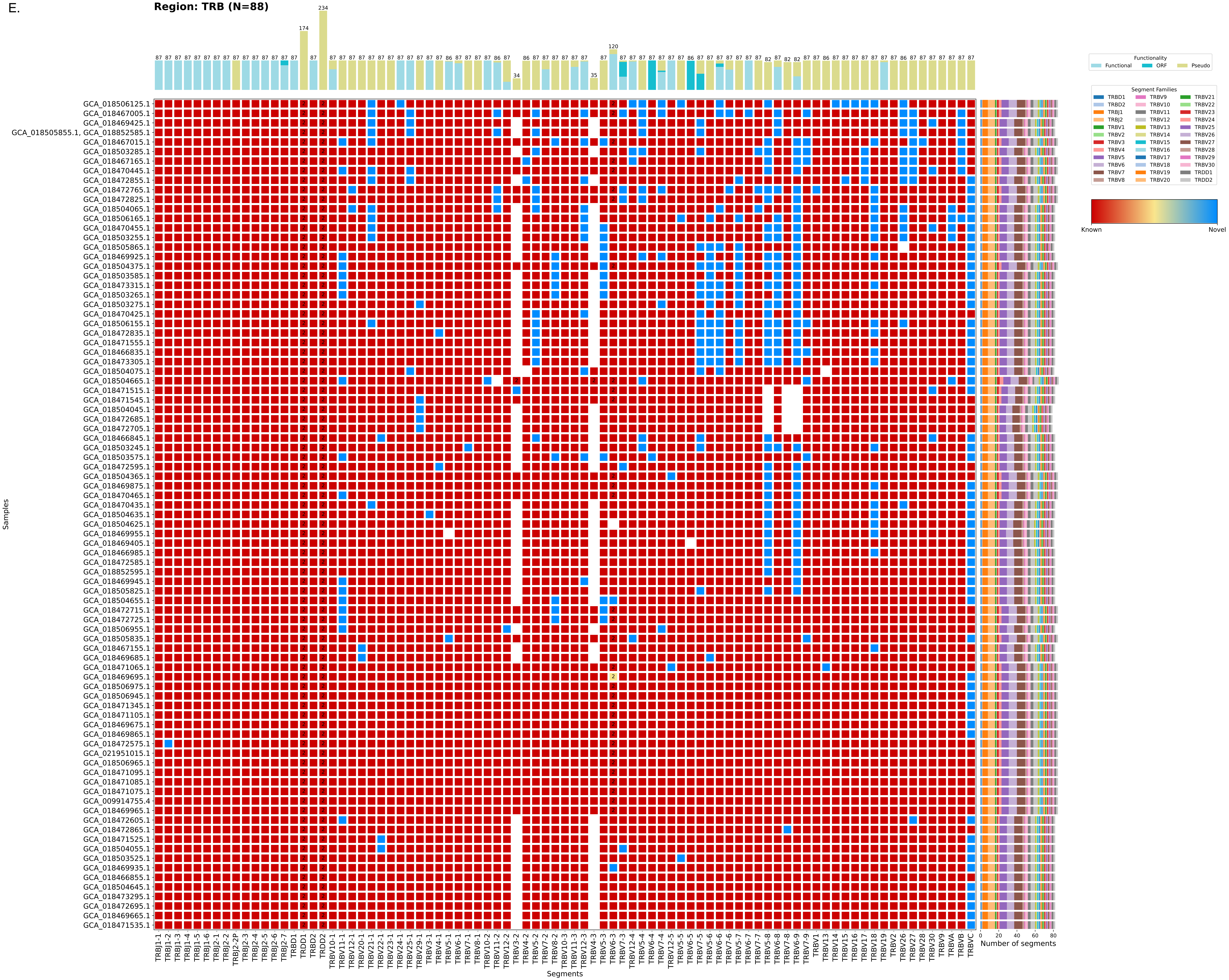
Region: TRB (N=88)
Functionality
Functional
ORF
Pseudo
Segment Families
Known
Novel
Samples
Segments
Number of segments

F.

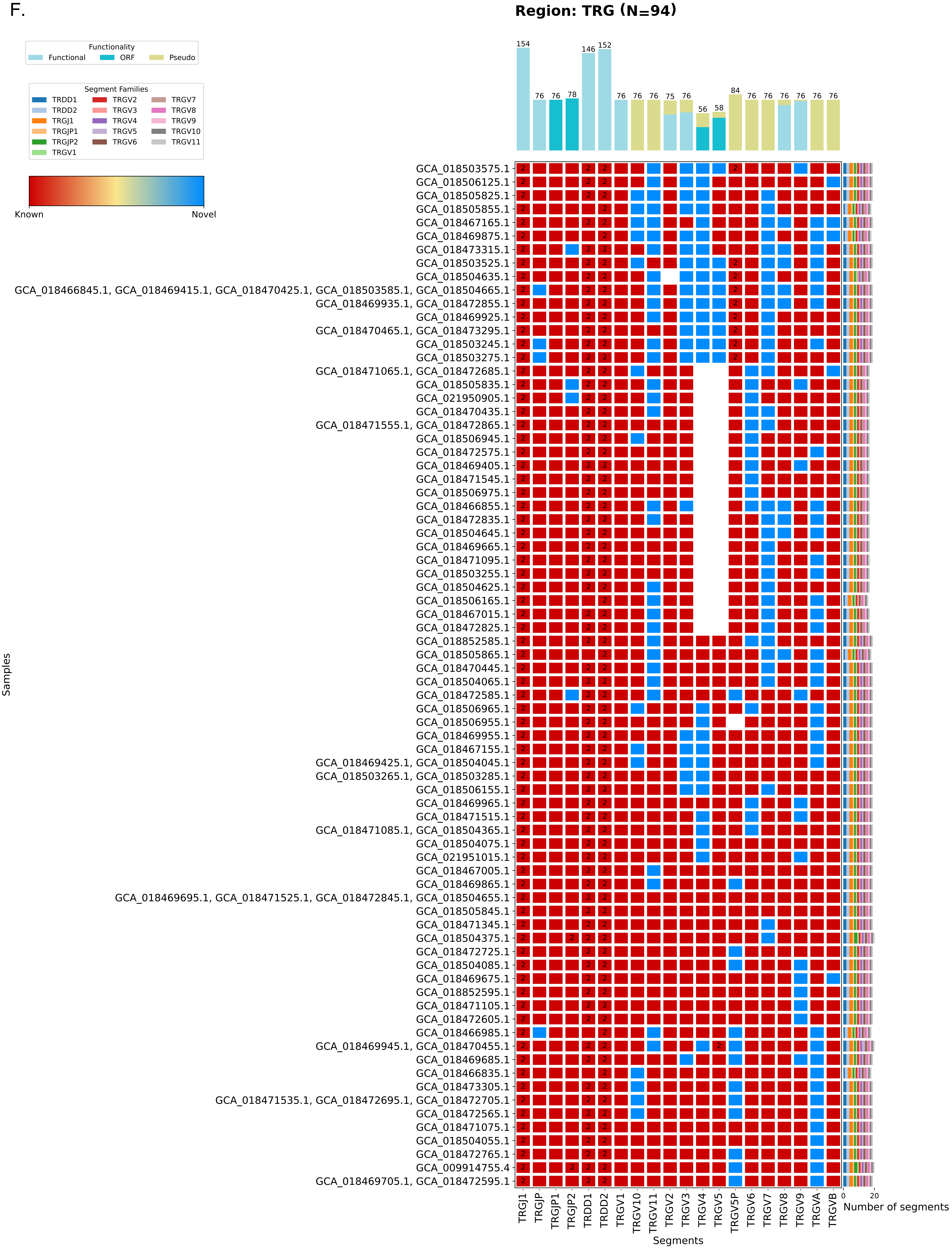
Region: TRG (N=94)
Functionality
Functional
ORF
Pseudo
Segment Families
TRDD1
TRDD2
TRGJ1
TRGJP1
TRGJP2
TRGV1
TRGV2
TRGV3
TRGV4
TRGV5
TRGV6
TRGV7
TRGV8
TRGV9
TRGV10
TRGV11
Known
Novel
154
76
76
78
146
152
76
76
76
75
76
56
58
84
76
76
76
76
76
76
Samples
GCA_018503575.1
GCA_018506125.1
GCA_018505825.1
GCA_018505855.1
GCA_018467165.1
GCA_018469875.1
GCA_018473315.1
GCA_018503525.1
GCA_018504635.1
GCA_018466845.1, GCA_018469415.1, GCA_018470425.1, GCA_018503585.1, GCA_018504665.1
GCA_018469935.1, GCA_018472855.1
GCA_018469925.1
GCA_018470465.1, GCA_018473295.1
GCA_018503245.1
GCA_018503275.1
GCA_018471065.1, GCA_018472685.1
GCA_018505835.1
GCA_021950905.1
GCA_018470435.1
GCA_018471555.1, GCA_018472865.1
GCA_018506945.1
GCA_018472575.1
GCA_018469405.1
GCA_018471545.1
GCA_018506975.1
GCA_018466855.1
GCA_018472835.1
GCA_018504645.1
GCA_018469665.1
GCA_018471095.1
GCA_018503255.1
GCA_018504625.1
GCA_018506165.1
GCA_018467015.1
GCA_018472825.1
GCA_018852585.1
GCA_018505865.1
GCA_018470445.1
GCA_018504065.1
GCA_018472585.1
GCA_018506965.1
GCA_018506955.1
GCA_018469955.1
GCA_018467155.1
GCA_018469425.1, GCA_018504045.1
GCA_018503265.1, GCA_018503285.1
GCA_018506155.1
GCA_018469965.1
GCA_018471515.1
GCA_018471085.1, GCA_018504365.1
GCA_018504075.1
GCA_021951015.1
GCA_018467005.1
GCA_018469865.1
GCA_018469695.1, GCA_018471525.1, GCA_018472845.1, GCA_018504655.1
GCA_018505845.1
GCA_018471345.1
GCA_018504375.1
GCA_018472725.1
GCA_018504085.1
GCA_018469675.1
GCA_018852595.1
GCA_018471105.1
GCA_018472605.1
GCA_018466985.1
GCA_018469945.1, GCA_018470455.1
GCA_018469685.1
GCA_018466835.1
GCA_018473305.1
GCA_018471535.1, GCA_018472695.1, GCA_018472705.1
GCA_018472565.1
GCA_018471075.1
GCA_018504055.1
GCA_018472765.1
GCA_009914755.4
GCA_018469705.1, GCA_018472595.1
TRGJ1
TRGJP
TRGJP1
TRGJP2
TRDD1
TRDD2
TRGV1
TRGV10
TRGV11
TRGV2
TRGV3
TRGV4
TRGV5
TRGV5P
TRGV6
TRGV7
TRGV8
TRGV9
TRGVA
TRGVB
Segments
0
20
Number of segments

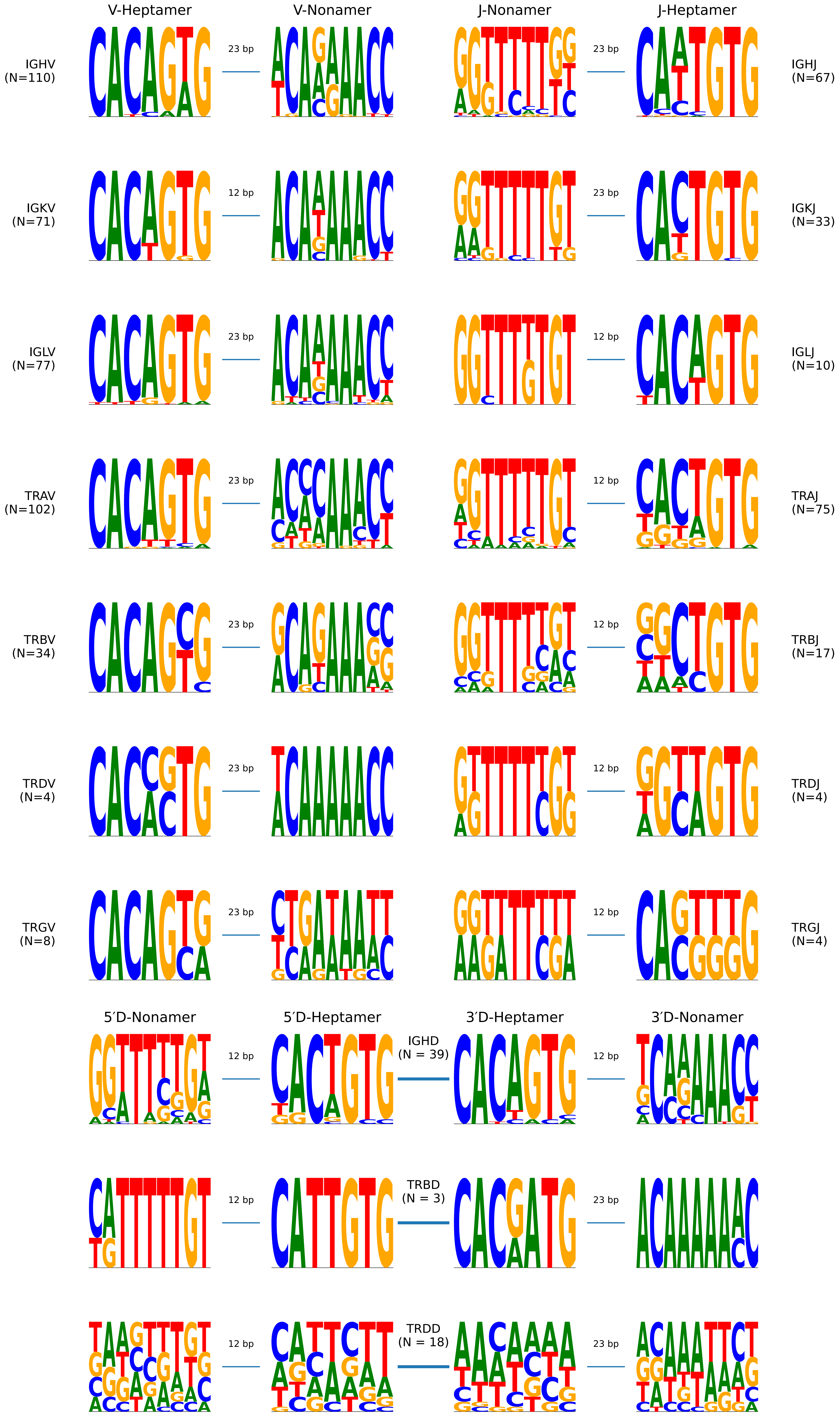

Supplemental figure 6. Recombination signal sequences (RSS) of V, D, and J gene seg-ments identified across human IG and TCR loci. Each row represents one locus, and the height of each symbol indicates the relative frequency of the corresponding nucleotide at each position. Every gene segment is flanked by a heptamer, followed by a spacer (12 or 23 bps), and a nonamer. D seg-ments display two RSS on both 5’ and 3’ sides. Only unique allele and RSS sequence combinations from functional gene segments (N) were in-cluded to generate these sequence motifs.

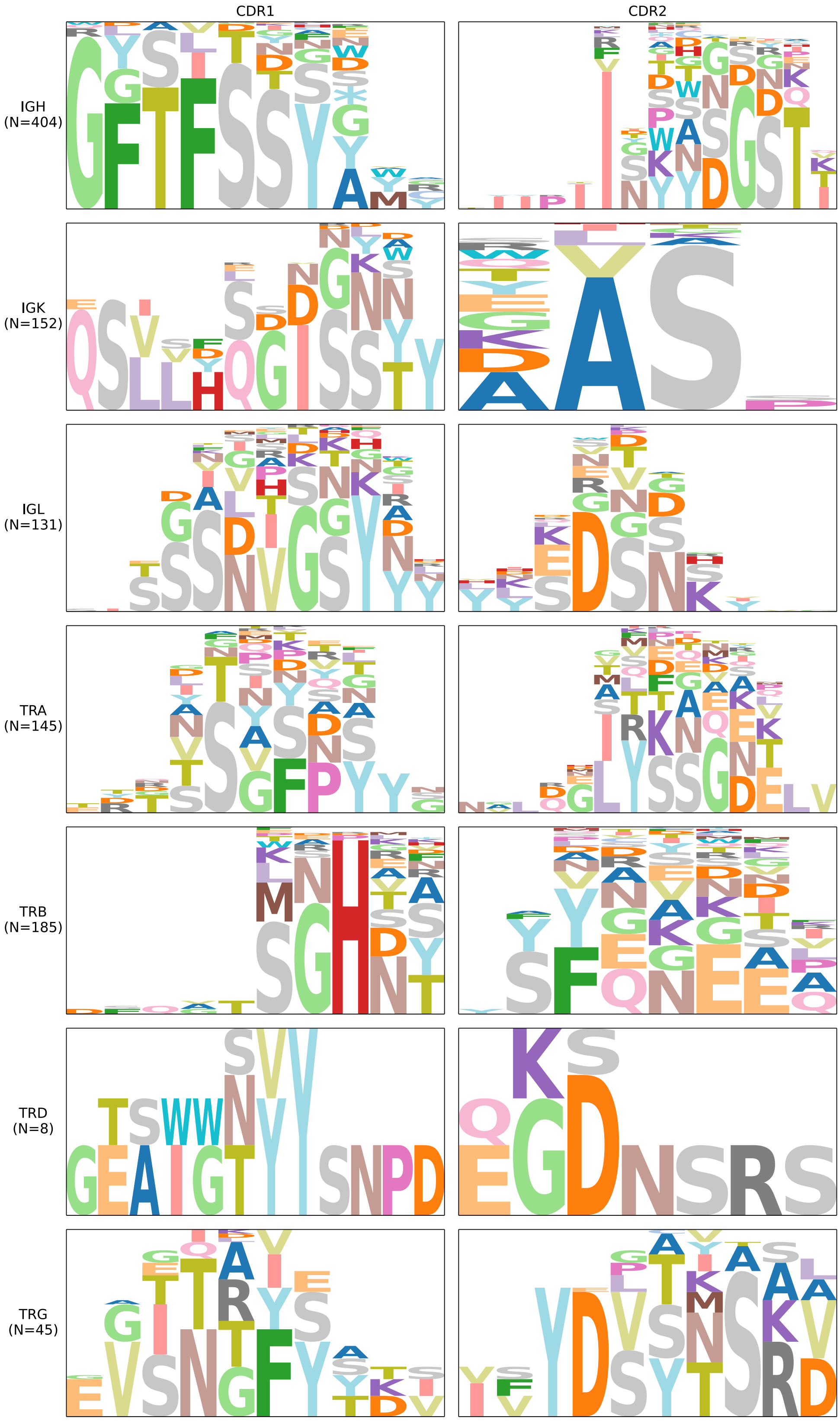

Supplemental figure S7. CDR1 and CDR2 sequence motifs across human IG and TCR loci. Each row represents a distinct locus, showing sequence logos for both CDR1 (left) and CDR2 (right). The height of each amino acid symbol indi-cates its relative frequency at that position within aligned sequences. Motifs were generated from alignments of CDR sequences of unique alleles (N) for each region. Positions where symbols do not reach the full height reflect alignment gaps, highlighting the variability in CDR lengths.
