# Supplementary material for: VDJ-Insights: simplifying the annotation of genomic immunoglobulin and T cell receptor regions
Source: Bioinformatics. 2026 Mar 9;42(4):btag108. doi: 10.1093/bioinformatics/btag108 (PMC13064985; doi:10.1093/bioinformatics/btag108)
Supplement: btag108_Supplementary_Data [file btag108_supplementary_data.zip › VDJ-Insights_Supplementary Methods_final.docx]

*Bioinformatics*, YYYY, 0–0

doi: 10.1093/bioinformatics/xxxxx

Advance Access Publication Date: DD Month YYYY

Manuscript Category

| Genome Analysis  VDJ-Insights: simplifying the annotation of genomic IG and TCR regions  Susan E. Ott^1^, Giang N. Le^1^, Sayed J. Mohammadi^1^, Jesse Mittertreiner^1^, Erica M. Pasini^1^, Ronald E. Bontrop^1^, Natasja G. de Groot^1^, and Jesse Bruijnesteijn^1^  ^1^ Biomedical Primate Research Centre, Lange Kleiweg 161, 2288 GJ Rijswijk, The Netherlands  Corresponding authors:  Jesse Bruijnesteijn \| bruijnesteijn@bprc.nl \| +31 15 2842 574  Susan Ott \| ott@bprc.nl \| +31 15 2842 792  Lange Kleiweg 161, 2288 GJ Rijswijk, The Netherlands  Associate Editor: XXXXXXX  Received on 06-08-2025; revised on XXXXX; accepted on XXXXX |
| --- |

# Supplementary Methods

**Details of the VDJ-Insights workflow**

VDJ-Insights accepts one or more assembled whole genomes, phased contigs, or pre-extracted target regions from any species as input and performs a comprehensive annotation analysis of the IG and TCR loci. The pipeline is organized into modular components, each responsible for a specific task in the workflow. These modules are executed via a Python-based driver script, which calls the required software tools for each step. VDJ-Insights adheres to recommended thread and memory settings specified by the tools it integrates. It supports multiprocessing by distributing available resources across samples within the same module, while reserving a 10% memory buffer to prevent system overload and ensure stable performance. Optional modules can be activated using command-line flags, such as the scaffolding of phased contigs prior to the identification and extraction of IG and TCR loci.

1. *Scaffolding of phased contigs (optional).* Discontinuous assemblies, often resulting from low sequencing coverage or extended homozygous regions, can cause the IG and TCR loci to be split across multiple contigs. To address this, VDJ-Insights offers an optional scaffolding step using RagTag (v2.1.0) (Alonge*et al.* 2022) with default settings, which aims to reconstruct more complete IG and TCR regions by aligning contigs to a reference sequence. This step can be activated using the -S flag in the command line, followed by the path to a reference FASTA file for scaffolding. Any immune loci annotated from scaffolded sequences will be flagged in the final annotation report, as these may still lack complete gene segment representation. VDJ-Insights also generates scaffold visualizations, showing which contigs were joined and how many gene segments were identified on each contig within the scaffold.
2. *Identification and extraction of IG and TCR regions.* To ensure annotation of the complete IG and TCR loci, VDJ-Insights first locates these regions using conserved flanking genes. For several model species, such as human, rhesus macaque, and mouse, these flanking genes are preconfigured as default settings within the tool (Suppl. Table S1). For other species, users can specify custom flanking genes via the command line in JSON format (e.g., -f '{"IGH": ["PACS2", "-"], "IGK": ["RPIA", "PAX8"], "IGL": ["GANZ", "TOP3B"]}'). If a locus is located near a telomeric chromosome end, as is the case with the human IGH region on chromosome 14, the absence of a distal flanking gene can be indicated using a hyphen ("-"). The sequences of all specified flanking genes are automatically retrieved using the NCBI Datasets command-line interface (CLI) (v15.25.0) (O'Leary*et al.* 2024).

The downloaded flanking gene sequences are aligned to the input assembly using minimap2 (v2.29) with asm5 settings (Li 2021). Based on these alignments, the corresponding genomic regions are extracted for downstream annotation using SAMtools (v1.22) (Li*et al.* 2009).

1. *IMGT scraping of gene segment libraries, leader sequences, and CDR sequences.* VDJ-Insights includes a custom Python-based scraping tool developed to retrieve data from the IMGT database (https://www.imgt.org). By using the -s flag followed by a species name (e.g., -s "Homo sapiens"), the script automatically downloads gene segment libraries, leader sequences, including L-Part1 and L-Part2, and complementarity-determining regions (CDR) 1 and 2 sequences. If leader or CDR sequences are not available for a given species, VDJ-Insight automatically omits functionality classification or CDR analysis.
2. *Annotation of V, D, and J gene segments.* By default, V, D, and J gene segment reference libraries are retrieved from the IMGT database. Alternatively, users may provide custom gene segment libraries in FASTA format (-l flag). Extracted IG and TCR regions were annotated by aligning the segment libraries using three independent mapping tools run in parallel: minimap2 (v2.29) (Li 2021) with parameters -a -m 70, Bowtie (v1.3.1) (Langmead*et al.* 2009) with parameters -k 5 -a -M 5 --strata, and Bowtie2 (v2.5.4) (Langmead and Salzberg 2012) with parameters --end-to-end --very-sensitive --score-min L,0,-0.5. The resulting alignment coordinates were converted to BED format using bedtools (v2.31.1) (Quinlan and Hall 2010) and used to extract the corresponding nucleotide sequences from the input assembly. To confirm the identity of mapped gene segments, all extracted sequences were re-aligned using the megablast algorithm in BLASTN (v2.14.1) (Chen*et al.* 2015). For sequences shorter than 50 bp, additional parameters were applied to improve sensitivity, including -penalty -3, -reward 1, -gapopen 5, -gapextend 2, and -word_size 7. BLAST output was parsed to retain only alignments with 100% query coverage. Gene segments with 100% identity to reference sequences in the library were classified as known. Segments containing mismatches, insertions, or deletions were classified as novel and subjected to further mutation analysis using BTOP (BLAST traceback operations) parsing (Camacho*et al.* 2009) to identify SNPs and indels at the nucleotide level.
3. *Prediction of gene segment functionality.* The functionality of identified V, D, and J gene segments was predicted using a defined set of criteria (Table 1). Briefly, after retrieving leader sequences, comprising L-PART1 and L-PART2, from the IMGT database, genomic flanking regions adjacent to annotated V and J gene segments were subsequently extracted based on coordinate data, accounting for strand orientation. To align the L-part sequences to these genomic regions, the blastn-short task from BLASTN (Chen*et al.* 2015) was employed with parameters optimized for short and high-sensitivity alignments: -word_size 7 -reward 1 -penalty -2 -gapopen 5 -gapextend 2 -best_hit_overhang 0.1 -best_hit_score_edge 0.1. Mapped coordinates from the BLAST output enabled reconstruction of full-length pre-mRNA transcripts by concatenating L-PART1, L-PART2, and the V-intron. Canonical splice motifs, donor (GT) and acceptor (AG) sites, were identified at intron-exon boundaries to validate splicing integrity. The reconstructed nucleotide sequences were then translated into protein using the standard genetic code, facilitating the evaluation of open reading frames (ORFs), identification of functional start codons, premature stop codons, and other conserved protein features. Finally, recombination signal sequence (RSS) annotations were integrated from the downstream step, enabling comprehensive functional classification of each gene segment.
4. *RSS analysis.* For each V, D, and J segment, flanking nucleotide sequences containing the RSS were extracted from the corresponding genomic FASTA files based on their strand orientation and known coordinates. Two sets of RSS sequences were generated: one containing all RSS candidates, and a second subset containing only RSS sequences that were temporarily classified as functional, using all non-RSS-related functionality criteria (Table 1). These latter sequences were used to build representative motif models. Motif discovery was performed using the MEME Suite (v5.5.8) (Bailey*et al.* 2015). The set of “temporarily” functional RSS sequences were input to MEME with the following parameters: -dna -mod zoops -nmotifs 1 and a fixed motif width equal to the expected RSS length. To detect motif occurrences across all extracted sequences, FIMO (Find Individual Motif Occurrences) (Grant, Bailey and Noble 2011) was run using the generated motif model and with all RSS sequences as input, with a search threshold of --thresh 0.0001 to maximize specificity and predict the final functionality. Segments lacking valid RSS motifs were reclassified as pseudogene.
5. *CDR analysis.* For each receptor type (IG or TCR) and segment class (e.g., IGHV, TRBV) genomic sequences were downloaded. The nucleotide sequences for CDR1 and CDR2 regions were extracted from each entry based on fixed positional offsets within the genomic sequences. Specifically, CDR1 was extracted from positions 78–113 and CDR2 from 165–194. These positions are based on established IMGT numbering conventions and were adjusted to remove gap characters (Lefranc 1997). To map CDR regions onto the target sequences, BLASTN (Chen*et al.* 2015) was used in short-read alignment mode (-task blastn-short). For each target, the top-scoring alignment was selected using a ranking strategy prioritizing query start position, alignment coverage, and match length.
6. *Annotation report.* To identify, filter, and annotate immune gene segments from high-throughput sequence alignments, a comprehensive data processing application is added to VDJ-Insights. This Flask application integrates alignment parsing, variant detection, gene segment classification, and standardized output generation (including BED, GTF, and Excel reports). The process distinguishes between known and novel gene segments and captures associated metadata for downstream interpretation including segment-based principal component analysis, dendrogram construction, and Venn diagram generation.

# References

Alonge M, Lebeigle L, Kirsche M *et al.* Automated assembly scaffolding using ragtag elevates a new tomato system for high-throughput genome editing. *Genome Biol* 2022;**23**:258. <https://doi.org/10.1186/s13059-022-02823-7>

Bailey TL, Johnson J, Grant CE, Noble WS. The meme suite. *Nucleic Acids Res* 2015;**43**:W39-49. <https://doi.org/10.1093/nar/gkv416>

Camacho C, Coulouris G, Avagyan V *et al.* Blast+: Architecture and applications. *BMC Bioinformatics* 2009;**10**:421. <https://doi.org/10.1186/1471-2105-10-421>

Chen Y, Ye W, Zhang Y, Xu Y. High speed blastn: An accelerated megablast search tool. *Nucleic Acids Res* 2015;**43**:7762-8. <https://doi.org/10.1093/nar/gkv784>

Grant CE, Bailey TL, Noble WS. Fimo: Scanning for occurrences of a given motif. *Bioinformatics* 2011;**27**:1017-8. <https://doi.org/10.1093/bioinformatics/btr064>

Langmead B, Salzberg SL. Fast gapped-read alignment with bowtie 2. *Nat Methods* 2012;**9**:357-9. <https://doi.org/10.1038/nmeth.1923>

Langmead B, Trapnell C, Pop M, Salzberg SL. Ultrafast and memory-efficient alignment of short DNA sequences to the human genome. *Genome Biol* 2009;**10**:R25. <https://doi.org/10.1186/gb-2009-10-3-r25>

Lefranc MP. Unique database numbering system for immunogenetic analysis. *Immunol Today* 1997;**18**:509. <https://doi.org/10.1016/s0167-5699(97)01163-8>

Li H. New strategies to improve minimap2 alignment accuracy. *Bioinformatics* 2021;**37**:4572-4. <https://doi.org/10.1093/bioinformatics/btab705>

Li H, Handsaker B, Wysoker A *et al.* The sequence alignment/map format and samtools. *Bioinformatics* 2009;**25**:2078-9. <https://doi.org/10.1093/bioinformatics/btp352>

O'Leary NA, Cox E, Holmes JB *et al.* Exploring and retrieving sequence and metadata for species across the tree of life with ncbi datasets. *Sci Data* 2024;**11**:732. <https://doi.org/10.1038/s41597-024-03571-y>

Quinlan AR, Hall IM. Bedtools: A flexible suite of utilities for comparing genomic features. *Bioinformatics* 2010;**26**:841-2. <https://doi.org/10.1093/bioinformatics/btq033>
